# Identification of Wee1 as a target in combination with avapritinib for gastrointestinal stromal tumor treatment

**DOI:** 10.1172/jci.insight.143474

**Published:** 2021-01-25

**Authors:** Shuai Ye, Dinara Sharipova, Marya Kozinova, Lilli Klug, Jimson D’Souza, Martin G. Belinsky, Katherine J. Johnson, Margret B. Einarson, Karthik Devarajan, Yan Zhou, Samuel Litwin, Michael C. Heinrich, Ronald DeMatteo, Margaret von Mehren, James S. Duncan, Lori Rink

**Affiliations:** 1 Molecular Therapeutics Program, Fox Chase Cancer Center, Philadelphia, Pennsylvania, USA.; 2 Pirogov Russian National Research Medical University, Moscow, Russia.; 3 Portland VA Health Care System and OHSU Knight Cancer Institute, Portland, Oregon, USA.; 4 Cancer Biology Program and; 5 Department of Biostatistics and Bioinformatics, Fox Chase Cancer Center, Philadelphia, Pennsylvania, USA.; 6 Department of Surgery, Perelman School of Medicine, University of Pennsylvania, Philadelphia, Pennsylvania, USA.

**Keywords:** Oncology, Therapeutics, Cancer

## Abstract

Management of gastrointestinal stromal tumors (GISTs) has been revolutionized by the identification of activating mutations in *KIT* and *PDGFRA* and clinical application of RTK inhibitors in advanced disease. Stratification of GISTs into molecularly defined subsets provides insight into clinical behavior and response to approved targeted therapies. Although these RTK inhibitors are effective in most GISTs, resistance remains a significant clinical problem. Development of effective treatment strategies for refractory GISTs requires identification of novel targets to provide additional therapeutic options. Global kinome profiling has the potential to identify critical signaling networks and reveal protein kinases essential in GISTs. Using multiplexed inhibitor beads and mass spectrometry, we explored the majority of the kinome in GIST specimens from the 3 most common molecular subtypes (*KIT* mutant, *PDGFRA* mutant, and succinate dehydrogenase deficient) to identify kinase targets. Kinome profiling with loss-of-function assays identified an important role for G_2_/M tyrosine kinase, Wee1, in GIST cell survival. In vitro and in vivo studies revealed significant efficacy of MK-1775 (Wee1 inhibitor) in combination with avapritinib in *KIT* mutant and *PDGFRA* mutant GIST cell lines as well as notable efficacy of MK-1775 as a monotherapy in the engineered *PDGFRA* mutant line. These studies provide strong preclinical justification for the use of MK-1775 in GIST.

## Graphical abstract

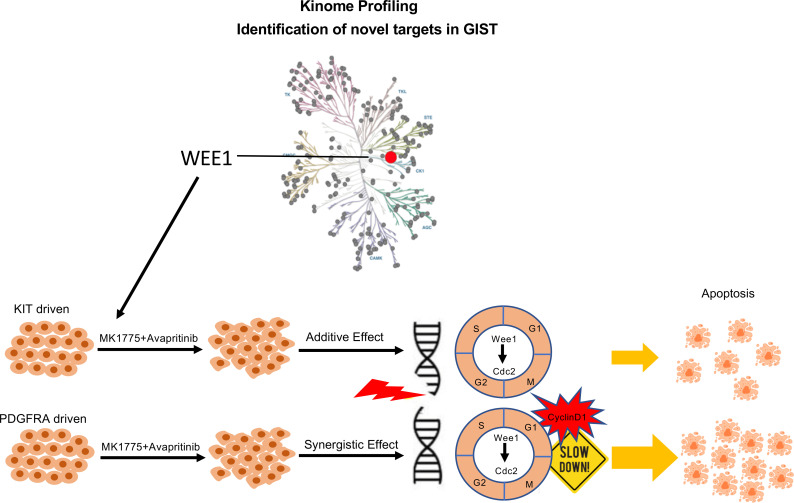

## Introduction

Gastrointestinal stromal tumors (GISTs) are the most common mesenchymal tumors of the gastrointestinal tract, with 5000–6000 new cases diagnosed annually in the United States (1). These tumors are characterized by near-universal expression of the RTK KIT, and the majority of GISTs harbor constitutively active mutant isoforms of KIT (70%–80%) or the related RTK, PDGFRA (5%–7%) (2). The approximately 10%–15% of GISTs that lack mutations in these genes often exhibit genetic or epigenetic deficiencies in the succinate dehydrogenase (SDH) complex of the respiratory chain (3, 4) and are referred to as SDH-deficient (SDH-d). Therapeutic targeting of GISTs with the frontline RTK inhibitor, imatinib mesylate (IM), along with 3 other FDA-approved agents (sunitinib, regorafenib, and ripretinib), has transformed therapy for advanced, unresectable GISTs. However, this “one-size-fits-all” approach to GIST treatment fails to address the molecular and clinical heterogeneity of these tumors. Tumor genotype has been shown to be an independent prognostic factor and a predictor of IM response in GISTs (5). The majority of GISTs harbor mutations in *KIT* that affect the juxtamembrane domain encoded by exon 11. Although tumors with mutations in this region generally initially respond well to IM therapy, they may exhibit negative prognostic features and aggressive biology (6). In contrast, *PDGFRA* mutant and SDH-d GISTs may exhibit a more indolent clinical course (7); however, the majority of these GIST subtypes demonstrate little or no response to IM (8, 9) or other approved therapies. The most common *PDGFRA* mutation found in GISTs, the D842V substitution, is particularly insensitive to IM. Avapritinib (BLU-285, Blueprint Medicines), a highly selective inhibitor of *KIT* exon 17 and *PDGFRA* exon 18 activation loop mutants, has demonstrated efficacy in vitro (10) and in vivo (11). Phase I testing (NAVIGATOR study, NCT02508532) has demonstrated notable efficacy for exon 18 *PDGFRA* mutant GIST (12), leading to FDA approval for the use of avapritinib in unresectable or metastatic *PDGFRA* exon 18 mutant GIST in January 2020.

Although these RTK inhibitors are effective in most GISTs, primary and acquired resistance remains a serious clinical obstacle. For clinical management of refractory GISTs to improve, new therapeutic targets must be identified. Finding a better way forward will require a more complete understanding of how the particular molecular aberrations in GIST subsets affect tumor signaling pathways and ultimately impact clinical behavior and therapeutic response. Differences in global gene expression and genomic profiles have been reported for GIST subtypes (3, 13–14); however, kinome profiling of GISTs has not been performed to date. Global kinome profiling has the potential to identify essential signaling networks and reveal protein kinases that are critical in GISTs. Protein kinases are highly druggable, with more than 45 FDA-approved kinase inhibitors (15), the majority of which are used clinically to treat malignancies. Several chemical proteomics approaches have been developed that measure levels of a large proportion of the kinome in cells and tissues, including Kinobeads, Kinativ, and multiplexed inhibitor beads and mass spectrometry (MIB-MS) (16–18). MIBs comprise a layered mixture of immobilized ATP-competitive pan-kinase inhibitors that enriches endogenous protein kinases from cell lysates based on affinity of individual kinases for the different immobilized inhibitors, their kinase abundance, and/or kinase activation state (17).

In this work, using MIB-MS (19, 20), we explored a high percentage (296 of 518) of the human kinome in treatment-naive primary GIST specimens from 3 GIST subtypes (*KIT* mutant, *PDGFRA* mutant, and SDH-d GISTs) to identify potential targets. Using this proteomics approach, we demonstrated that the 3 GIST subtypes have distinct kinome profiles and identified kinases that are universally overexpressed in all GISTs as well as kinases that are unique to each subtype. Finally, kinome profiling in combination with loss-of-function validation assays revealed an important role for the G_2_/M tyrosine kinase, Wee1, in GIST survival. We also report significant efficacy of MK-1775 (Wee1 inhibitor) as a monotherapy and in combination with avapritinib in an engineered GIST cell line driven by an activating *PDGFRA* D842V mutation. The combination was also effective in controlling the growth of these PDGFRA-driven GIST cells in three-dimensional spheroid culture. Furthermore, dual inhibition of Wee1 and KIT/PDGFRA in GIST xenografts provided disease stabilization and improved survival.

## Results

### Kinome profiling of primary GIST using MIB-MS.

**Figure 1:**
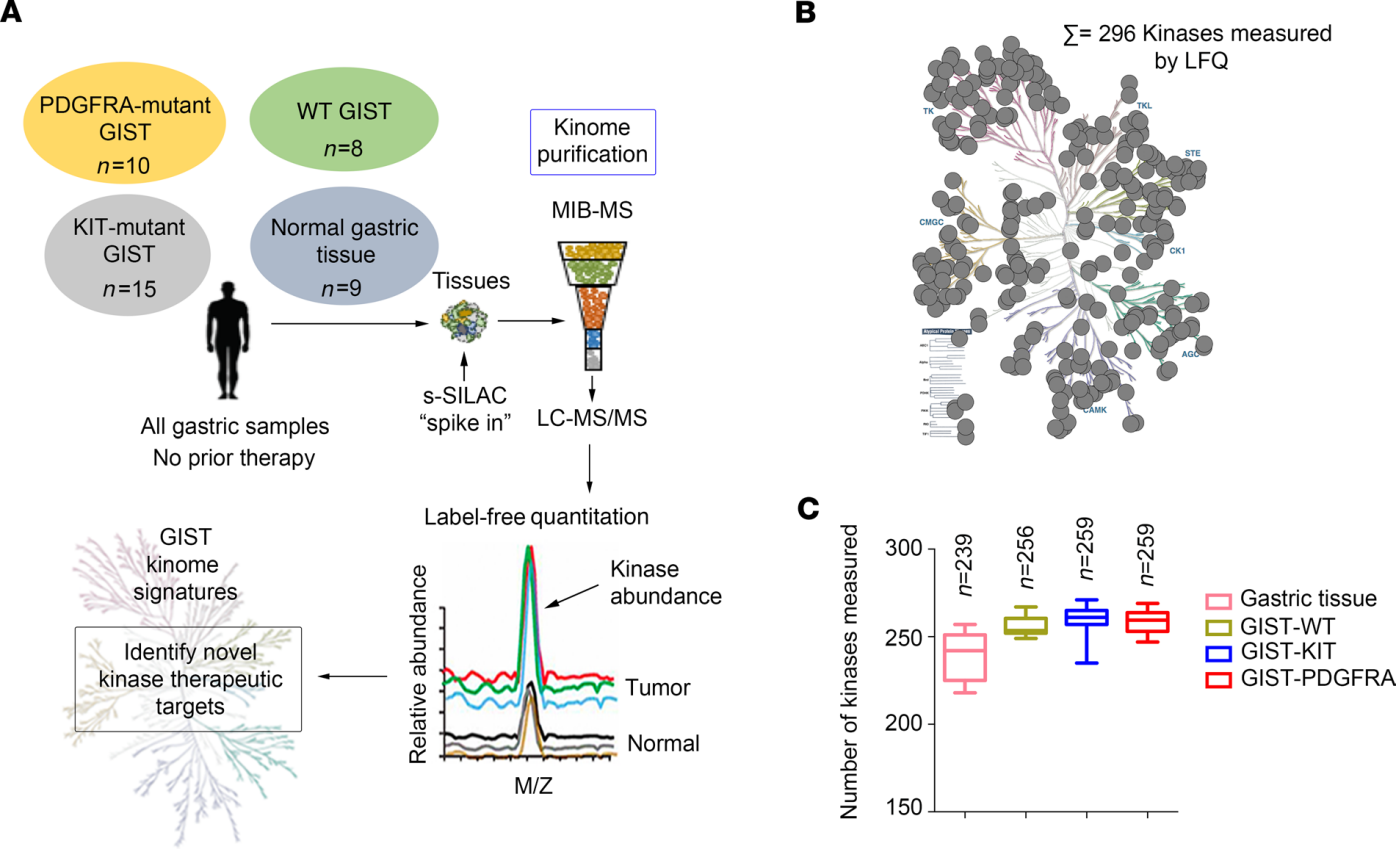
Characterizing the GIST kinome in primary tumors using MIB-MS to identify therapeutic targets. (**A**) Schematic of experimental approach. MIB-MS was used to quantify the kinase abundance in patients with GISTs (untreated, gastric primary GIST from 3 molecular subtypes: *KIT* mutant, *n* = 15; *PDGFRA* mutant, *n* = 10; WT GIST, *n* = 8; and normal gastric tissue, *n* = 9) to map the proteomic landscape of the kinome and identify targets. Kinase levels in tissues were determined using a combination of LFQ and s-SILAC. (**B**) Kinome tree depicts fraction of kinome quantitated by MIB-MS and frequency across 42 samples measured. (**C**) Average number of kinases detected by MIB-MS profiling broken down by tissue type. GIST, gastrointestinal stromal tumor; MIB-MS, multiplexed inhibitor beads and mass spectrometry; LFQ, label-free quantitation; s-SILAC, super-SILAC.

**Figure 2:**
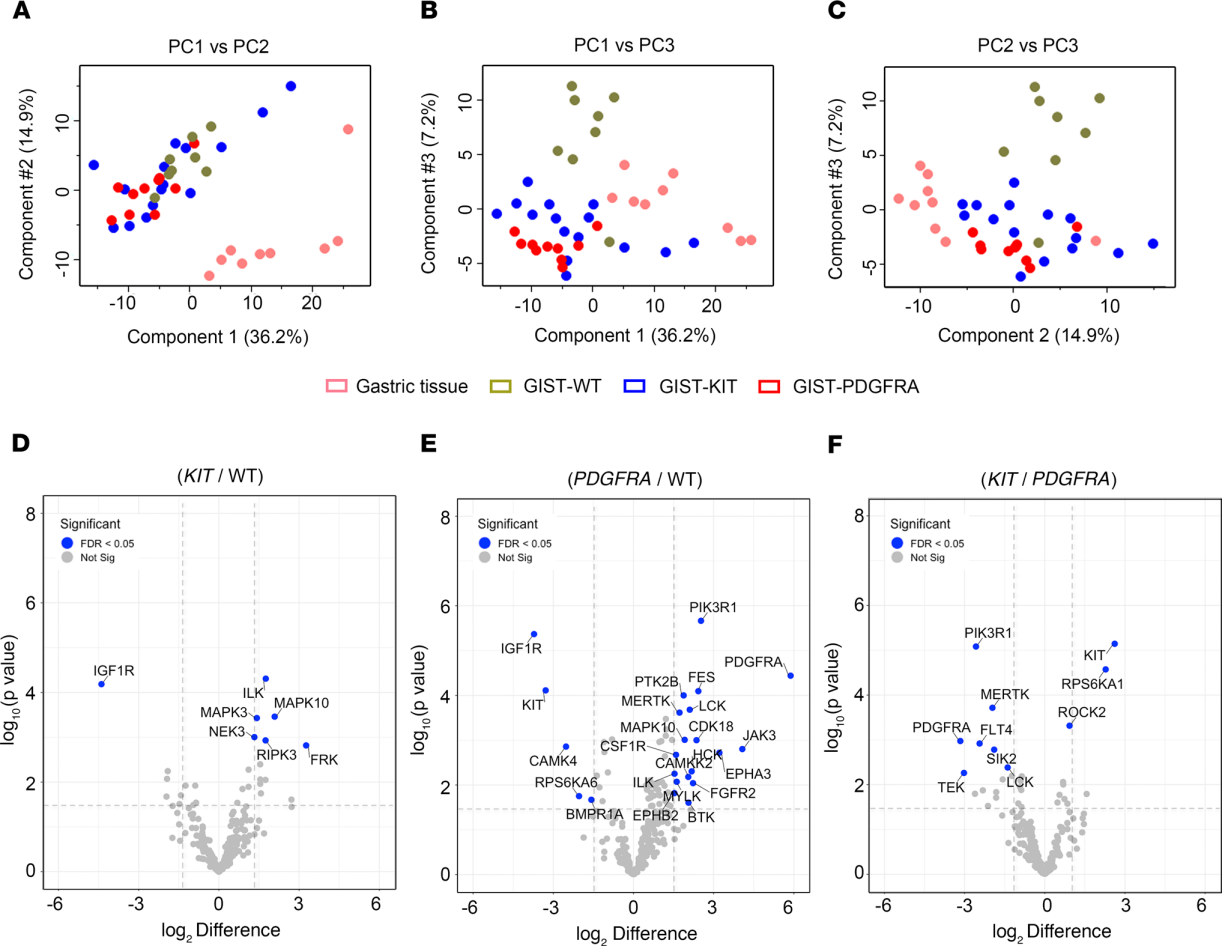
Mapping the distinct kinome signatures among GIST subtypes. (**A–C**) PCA, including PC1 vs. PC2 (**A**), PC1 vs.PC3 (**B**), and PC2 vs.PC3 (**C**) of MIB-MS in 3 GIST subtypes (*KIT* mutant, blue; *PDGFRA* mutant, red; WT, green) and normal gastric tissue (pink). (**D**) Volcano plot comparisons of KIT mutant vs. WT, (**E**) *PDGFRA* mutant vs. WT, and (**F**) *KIT* mutant vs. *PDGFRA* mutant GIST MIB-MS kinome profiles. Differences in kinase log2 LFQ intensities among tumors and normal tissues determined by paired *t* test Benjamini-Hochberg adjusted *P* values at FDR of <0.05 using Perseus software. PCA, principal component analysis; GIST, gastrointestinal stromal tumor; MIB-MS, multiplexed inhibitor beads and mass spectrometry; LFQ, label-free quantitation.

To explore the kinome landscapes among the 3 molecular subtypes of GIST, we performed MIB-MS profiling on 33 IM-naive primary gastric GIST specimens, which included the following subtypes: (a) *KIT* exon 11 mutants (*n* = 15), (b) *PDGFRA* mutants (*n* = 10), and (c) *KIT/PDGFRA*-WT GISTs (*n =* 8) (Table 1). The *KIT/PDGFRA*-WT GISTs include 7 SDH-d and 1 GIST that lacked *SDH* mutations and was shown by SDHB immunohistochemistry to have an intact SDH complex (21). We also kinome profiled 9 normal gastric tissues from donors without a history of kinase inhibitor therapy. To quantify the MIB-bound kinome of GIST tissues, we performed label-free protein quantitation (LFQ) using the MaxLFQ algorithm (22) in combination with a super-SILAC (s-SILAC) (23) internal standard to control for variations in kinase MIB-binding and/or liquid chromatography–tandem MS (LC-MS/MS) retention time reproducibility (Figure 1A). In total, we measured MIB-binding values for 296 kinases across these GIST samples, with 242 kinases quantitated in greater than 70% of tissues profiled and 156 kinases measured in every MIB-MS run. The average number of kinases measured for each sample was 254 (Figure 1, B and C, and Supplemental Data file 1; supplemental material available online with this article; https://doi.org/10.1172/jci.insight.143474DS1). Principal component analysis (PCA) and hierarchical clustering of MIB-MS profiles revealed that the GIST kinome is overall distinct from normal gastric tissues (Figure 2A and Supplemental Figure 1). Furthermore, PCA of the kinome profiles revealed *KIT* mutant and *PDGFRA* mutant GISTs grouped distinctly from KIT/PDGFRA-WT GISTs (Figure 2, B and C). One exception to this was the *KIT/PDGFRA*-WT33 sample, which clustered closer to *KIT* mutant GIST samples. Interestingly, this sample was distinct from other KIT/PDGFRA-WT GIST samples in that it possessed an intact SDH complex and most likely has an unknown driver mutation.

### Mapping the distinct kinome signatures among GIST subtypes.

Volcano plot analysis of kinase log_2_ LFQ values (Figure 2, D–F) revealed kinases exhibited differential protein abundance among GIST subtypes (Supplemental Data file 1). A scatter plot comparing LFQ- or s-SILAC–determined log2 differences in kinases levels among tissues showed that the 2 quantitative methods displayed substantial overlap, validating the majority of kinase measurements (Supplemental Figure 2, A–F). Some kinases were not quantitated by the s-SILAC method, which can be attributed to low or absent expression of kinase in SILAC-labeled cell line cocktail. IGF1R was the top-ranking kinase elevated in *KIT/PDGFRA*-WT versus *KIT* mutant or *PDGFRA* mutant subtypes, as previously described (24, 25) (Figure 2, D and E, and Supplemental Figure 1, B–D). *PDGFRA* mutant tumor kinome profiles greatly differed from *KIT/PDGFRA*-WT tumors, with many kinases showing increased protein levels in PDGFRA mutant tumors relative to WT (Figure 2E and Supplemental Figure 1D). Elevated levels of several RTKs, including PDGFRA, MERTK, EPHB2, EPHA3, CSF1R, and FGFR2, were observed in *PDGFRA* mutant tumors versus WT tumors, as well as increased levels of PIK3R1, the regulatory subunit of PIK3CA, which has been shown to be an important downstream signaling effector of both KIT and PDGFRA (26) (Figure 2E and Supplemental Figure 2, C and D). Notably, many of the elevated kinases in the *PDGFRA* mutant tumors were kinases related to immune cell function, including HCK, LCK, BTK, CSF1R, and MERTK. These findings are consistent with a recent report from Vitiello et al. (27) demonstrating increased immune cells present in *PDGFRA* mutant GISTs. Conversely, increased ROCK2 was detected in *KIT* mutant versus *PDGFRA* mutant tumors (Figure 2F and Supplemental Figure 2F). ROCK2 has been associated with increased aggressiveness and metastasis, as well as poor overall survival in several malignancies (28).

### Targeting the GIST kinome signature identified WEE1 as candidate target.

**Figure 3:**
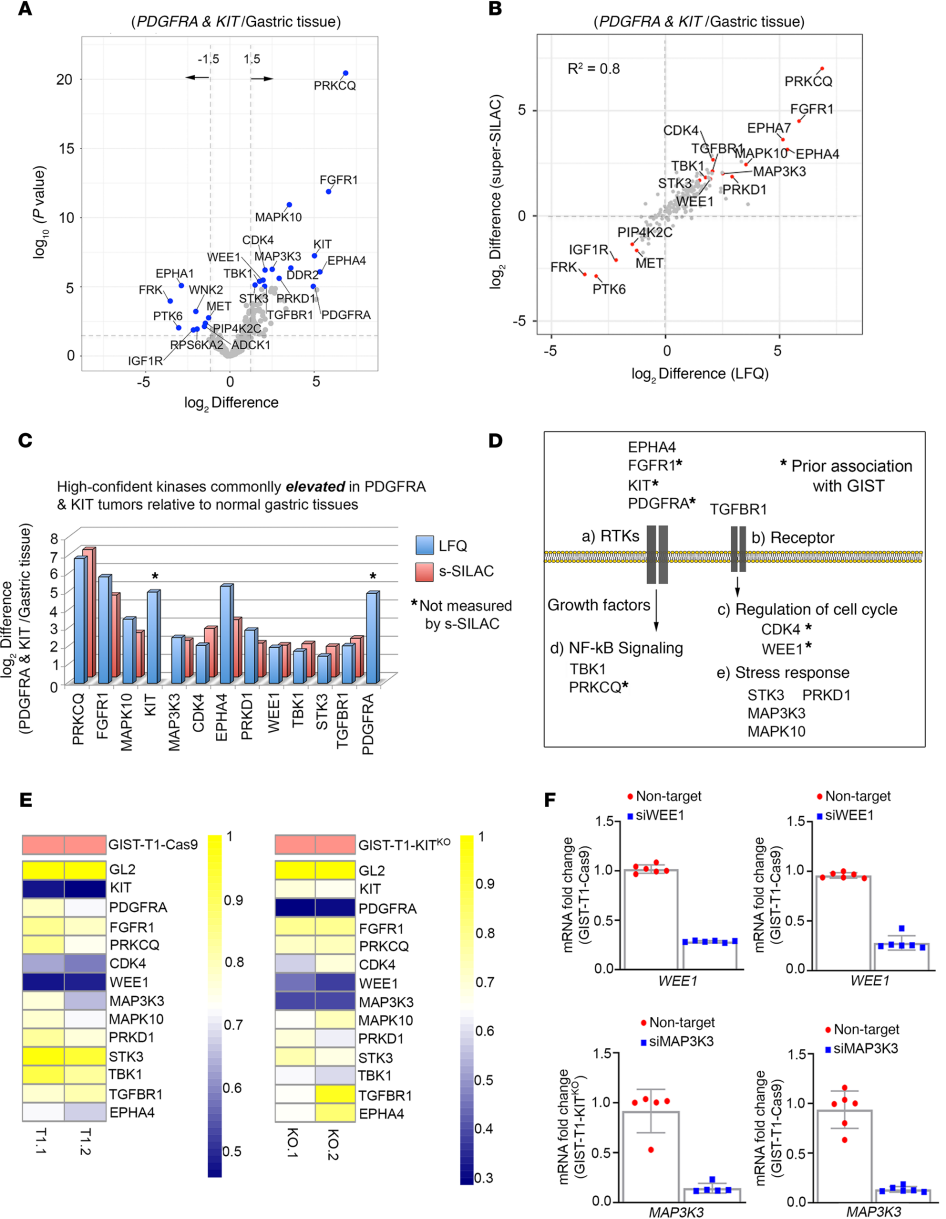
Targeting the mutant-GIST kinome signature identifies WEE1 as candidate target. (**A**) Volcano plot comparisons of *KIT* mutant and *PDGFRA* mutant GIST vs. normal gastric tissue MIB-MS kinome profiles. Differences in kinase log2 LFQ intensities among tumors and normal tissues determined by paired *t* test Benjamini-Hochberg adjusted *P* values at FDR <0.05 using Perseus software. (**B**) Scatter plot depicts overlap in kinases elevated or reduced determined by LFQ or s-SILAC. Regression analysis (R^2^) among quantitative methods was performed in Perseus software. Differential expressed kinases commonly identified by LFQ and s-SILAC quantitation (FDR <0.05) are labeled. (**C**) Bar graph depicts high-confident kinases log2 LFQ *z* scores overexpressed in mutant-GIST determined by LFQ and/or s-SILAC quantitation (FDR <0.05). (**D**) Associated pathways/functions of kinases overexpressed in *KIT* mutant and *PDGFR*A mutant GIST vs. normal tissues determined by quantitative MIB-MS profiling. (**E**) Heatmap depicting viability scores for siRNA library screen targeting high-confident kinases elevated in *KIT* mutant and *PDGFRA* mutant GIST in GIST-T1+Cas9 and GIST-T1+D842V KIT^KO^ cell lines as measured by Cell Titer Blue assay. siGL2 was negative control, viability score = 1.0. Two independent replicates were performed per cell line. (**F**) Quantitative RT-PCR confirmed >70% knockdown of Wee1 (top) and MAP3K3 (bottom) mRNA in both cell lines. Expression levels were normalized to HPRT. Data represent mean ± SD. GIST, gastrointestinal stromal tumor; MIB-MS, multiplexed inhibitor beads and mass spectrometry; LFQ, label-free quantitation; s-SILAC, super-SILAC.

Next, we explored kinases commonly overexpressed among *KIT* mutant and *PDGFRA* mutant tumors relative to normal gastric tissue, with the goal of identifying kinase targets to exploit in GISTs. As expected, volcano plot analysis showed significant differences between normal gastric tissue and GIST mutant tumors, with numerous kinases expressed at higher levels in *KIT* mutant and *PDGFRA* mutant tumors relative to normal gastric tissues (Figure 3A and Supplemental Data file 1). The majority of LFQ-determined kinase measurements were confirmed by s-SILAC, representing high-confidence kinase signatures (Figure 3B). Elevation of KIT, PRKCQ, and FGFR1, all of which have been previously shown to be upregulated in GISTs (29, 30), as well as kinases associated with regulation of cell cycle (WEE1 and CDK4), NF-kB signaling (TBK1), and stress response signaling (MAP3K3, STK3, MAPK10 and PRKD1) (Figure 3, C and D) was seen. To explore functional relevance of the high-confidence kinases commonly elevated in *KIT* mutant and *PDGFRA* mutant tumors, we designed a kinase-centric siRNA library to identify kinases that are critical for *KIT* mutant and *PDGFRA* mutant GIST cell survival. This siRNA library contained pooled siRNAs targeting each of the 13 kinases identified in the kinome profiling experiment. Synthetic lethal screens were performed using an isogenic pair of cell lines: GIST-T1+Cas9 (KIT driven) and GIST-T1+D842V KIT^KO^ (PDGFRA D842V driven). Positive controls for the screen included siKIT (GIST-T1+Cas9) and siPDGFRA (GIST-T1+D842V KIT^KO^), whereas siGL2 served as negative control for both lines. Knockdown of the majority of the kinases in the screen showed minimal impact on cell viability (Figure 3E). However, siRNA-mediated depletion of WEE1 led to significantly decreased viability in both isogenic lines (viability score = 0.48 in GIST-T1+Cas9; 0.41 in GIST-T1+D842V KIT^KO^), whereas knockdown of MAP3K3 affected viability in the *PDGFRA* mutant cell line (viability score = 0.39). Viability reductions approaching that of the KIT and PDGFRA positive controls were seen for these 2 kinases. Greater than 70% Wee1 knockdown was achieved in both cell lines (Figure 3F). Interestingly, depletion of MAP3K3, known to promote ovarian and NSCLC tumor growth (31, 32), inhibited *PDGFRA* mutant GIST cell viability; however, no selective small molecule inhibitors are currently available to explore targeting MAP3K3 in GISTs. Overexpression of Wee1 has also been observed in numerous malignancies, including breast and melanoma (33). MK-1775 (adavosertib, AZD1775), a selective inhibitor targeting Wee1, is under investigation in clinical trials, and, recently, several preclinical studies have suggested synergy when Wee1 inhibitors were combined with other kinase inhibitors, including the mTOR inhibitor, TAK228 (34), and the AURKA inhibitor, alisertib (35). Our GIST kinome profiling data along with these initial cell viability studies suggest that Wee1 could be a plausible drug target in mutant GIST, either alone or in combination with existing therapies.

### MK-1775 and avapritinib had enhanced combination effects on in vitro GIST cell growth.

**Figure 4:**
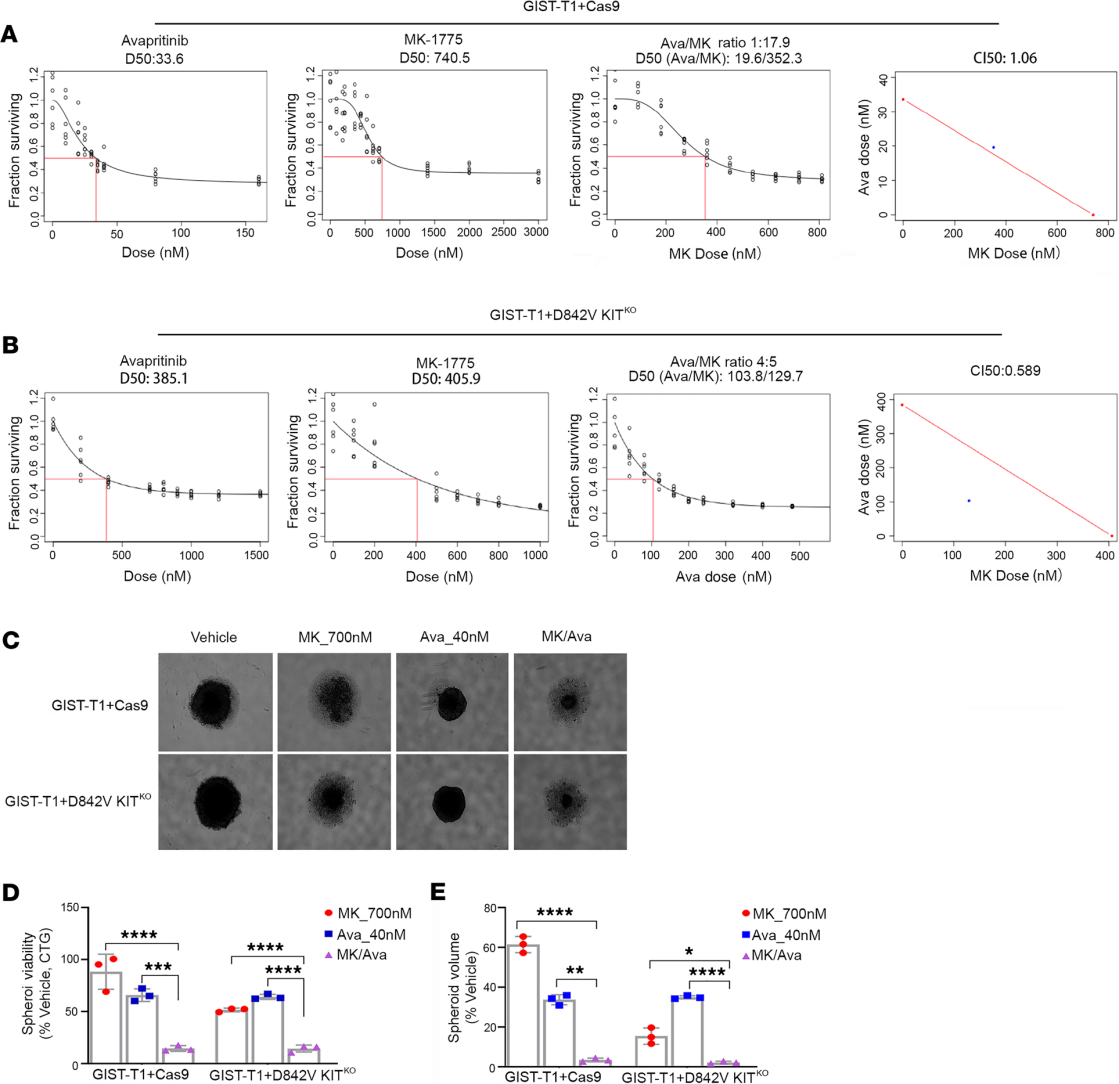
MK-1775 and avapritinib have enhanced combination on in vitro GIST cell growth. Panels 1 and 2 show dose response curves for single agents (avapritinib, MK-1775) in GIST-T1+Cas9 (**A**) and GIST-T1+D842V KIT^KO^ (**B**) cell lines. Red box indicates estimation of LD50 concentration for each single drug. Panel 3 shows dose response curve representing increasing series of combinations in GIST-T1+Cas9 (**A**) and GIST-T1+D842V KIT^KO^ (**B**) cell lines. Red box indicates estimation of LD50 concentration for combination of drugs. Panel 4 shows single point (blue) on isobole curve for 50% kill. Red line indicates 50% isobole for strictly additive effect. CI_LD50_ in GIST-T1+Cas9 is 1.06 and not found in the synergistic triangle (region below the red line) (**A**). CI_LD50_ is 0.589 in GIST-T1+D842V KIT^KO^ and is found within the synergistic triangle (**B**). Representative images of GIST-T1+Cas9 and GIST-T1+D842V KIT^KO^ spheroids after 120-hour treatment at indicated concentrations (**C**). Bars represent average viability ± SEM after 120-hour treatment at indicated drug concentrations for GIST-T1+Cas9 and GIST-T1+D842V KIT^KO^ spheroids as a percentage of vehicle-treated spheroids (**D**). Bars represent the average spheroid volume ± SEM of GIST-T1+Cas9 and GIST-T1+D842V KIT^KO^ spheroids as a percentage of vehicle-treated spheroids (**E**). All spheroid data were analyzed using GraphPad Prism, with comparisons of treatment groups performed in 1-way ANOVA and post hoc comparisons made using Bonferroni’s multiple comparisons method; *P = 0.0165, **P = 0.0046, ***P = 0.0008, ****P ≤ 0.0001. GIST, gastrointestinal stromal tumor.

Although avapritinib has demonstrated dramatic responses in *PDGFRA* mutant GISTs harboring the D842V mutation, acquired resistance to this monotherapy has been observed. A large body of evidence suggests that targeting multiple tumor signaling pathways simultaneously may lead to more sustained tumor control. Given the promising kinome profiling data that demonstrated increased Wee1 activation in GISTs (Figure 3, B and C) and the significant effect on cell viability associated with Wee1 knockdown (Figure 3E), we tested the effects of combined inhibition of Wee1 using MK-1775, a commercially available selective inhibitor of Wee1, with KIT/PDGFRA inhibition using avapritinib. We evaluated the effects of MK-1775 and avapritinib using the GIST-T1+Cas9 (KIT driven) and GIST-T1+D842V KIT^KO^ (PDGFRA driven) cell lines, as single agents and in combination at increasing molar ratios. Figure 4, A and B, shows single-agent, dose-response curves for GIST-T1+Cas9 and GIST-T1+D842V KIT^KO^, respectively. We first estimated the LD50 for each agent in the 2 cell lines (Figure 4, A and B, left panels). We then treated each line with increasing doses of the 2 drugs in a fixed ratio as their LD50s (Figure 4, A and B, third panel). To quantify synergy, combination index (CI) values were calculated (Figure 4, A and B, last panel: CI values less than 1 are considered synergistic). The CI_LD50_ values for GIST-T1+Cas9 and GIST-T1+D842V KIT^KO^ were 1.06 and 0.589, respectively, indicating possible synergy only in the PDGFRA-driven cell line, which was then established to be significant via a bootstrap statistic (36). Although synergy was not observed in the GIST-T1+Cas9 cell line, a clear additive effect of the 2 drugs was detected. We also evaluated the in vitro effect of MK-1775 and avapritinib, alone and in combination, on a second KIT-driven, GIST882 cell line. This cell line has an ATP-binding site mutation in *KIT* exon 13. Previously reported biochemical data evaluating the activity of avapritinib against a spectrum of *KIT* and *PDGFRA* mutations indicated inferior activity in exon 13 *KIT* mutants compared with exons 11, 17, and 18 of KIT and *PDGFRA* D842V mutation (10). Supplemental Figure 3A shows single-agent, dose-response curves (panels 1 and 2) and combination (panel 3) in this cell line. As expected, avapritinib is less effective in GIST882 than in GIST-T1. However, the CI_LD20_ value was 0.237 (last panel), indicating possible synergy, which was established to be significant using a bootstrap statistic.

To evaluate the effects of the drugs as monotherapies or in combination on three-dimensional (3D) GIST cell growth, spheroid assays were performed, which more accurately mimic tumor physiology than cells grown in monolayer. GIST-T1+Cas9, GIST-T1+D842V KIT^KO^, and GIST882 cells form dense, uniformly spherical cultures with true cell-to-cell contacts that are maintained upon physical manipulations, indicative of true spheroids (Figure 4C and Supplemental Figure 3B). Treatment of both GIST-T1+Cas9 and GIST-T1+D842V KIT^KO^ spheroids with either of the single agents, MK-1775 (700 nM) or avapritinib (40 nM), resulted in decreased spheroid viability (Figure 4D) and volume (Figure 4, E and C) relative to vehicle-treated spheroids (Figure 4C). However, treatment of these spheroids with the combination resulted in a significantly greater reduction in both viability and volume (Figure 4, C–E). Interestingly, both MK-1775 as a monotherapy and in combination with avapritinib had greater efficacy in GIST-T1+D842V KIT^KO^ compared with GIST-T1+Cas9 spheroids. Given that avapritinib is known to have less efficacy against exon 13 *KIT* mutations, we subjected GIST882 spheroids to a higher dose of avapritinib (120 nM). Treatment of GIST882 spheroids with the combination of MK-1775 and avapritinib resulted in significantly greater reduction in both viability (Supplemental Figure 3C, left panel) and spheroid volume (Supplemental Figure 3C, right panel) compared with either single agent.

### Combination treatment increased DNA damage and apoptosis.

**Figure 5:**
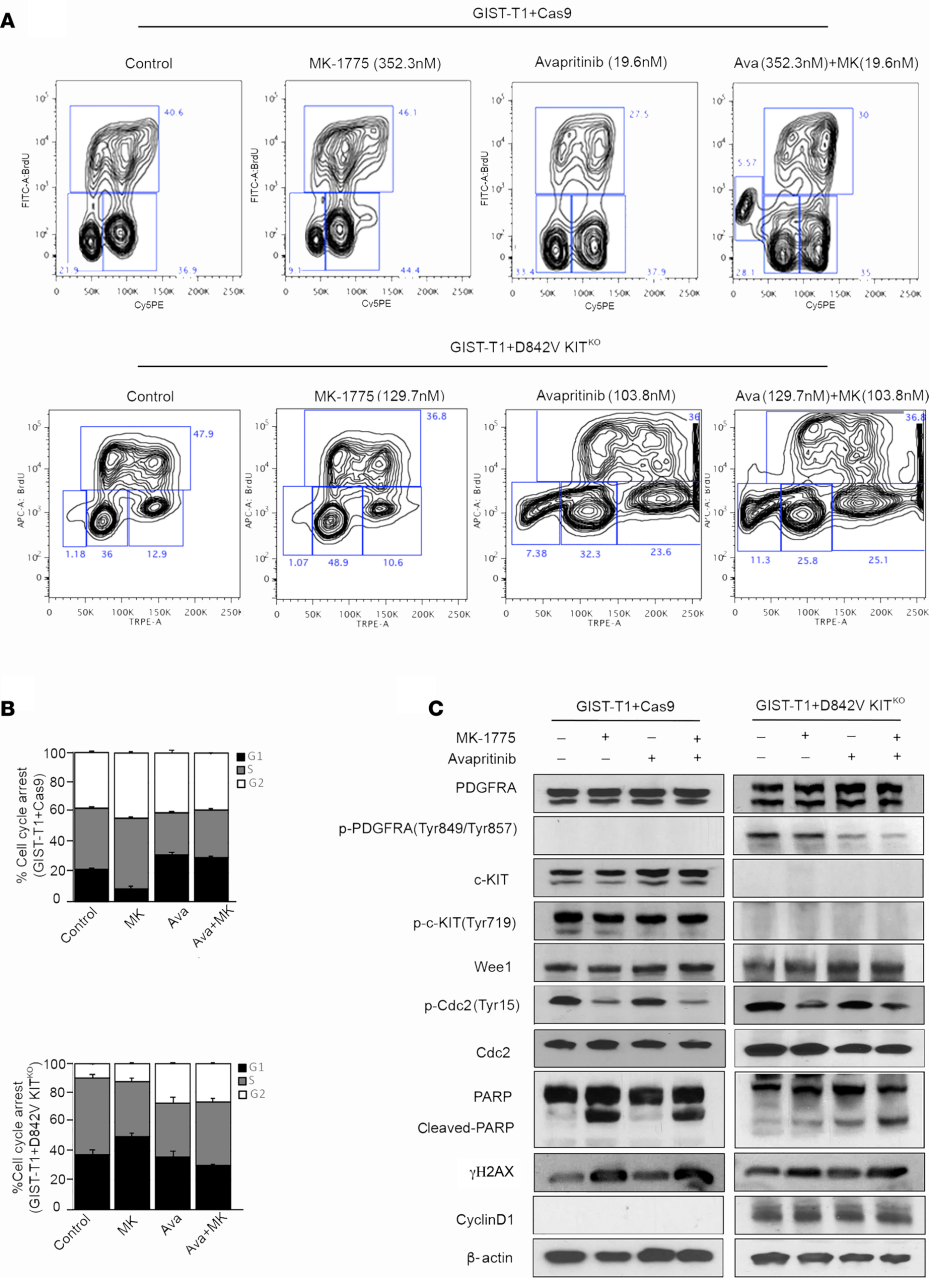
Mechanism of MK-1775 and avapritinib combination in KIT-dependent and –independent GIST cell lines. (**A**) Representative flow cytometry plots and (**B**) quantification of BrdU incorporation in GIST-T1+Cas9 (upper panel) treated with 352.3 nM MK-1775, 19.6 nM avapritinib and combination for 72 hours. Statistically significant differences were observed between the following comparisons: for G1 arrest, vehicle vs. avapritinib (*P* < 0.0009) and vehicle vs. avapritinib/MK-1775 (*P* < 0.0002); for G_2_ arrest, vehicle vs. MK-1775 (*P* < 0.005). (**A**) Representative flow cytometry plots and (**B**) quantification of BrdU incorporation in GIST-T1-D842V+ KIT^KO^ treated (bottom panel) with 129.7 nM MK-1775, 103.8 nM avapritinib and combination for 72 hours. Statistically significant differences were observed between the following comparisons: for G1 arrest, vehicle vs. MK-1775 (*P* < 0.0001); for G_2_ arrest, vehicle vs. avapritinib (*P* < 0.005), vehicle vs. avapritinib/MK-1775 (*P* < 0.0002). Data represent mean ± SD. (**C**) Immunoblot assays of WCEs from GIST-T1+Cas9 (KIT-dependent) and GIST-T1+D842V KIT^KO^ (KIT-independent) cell lines treated as in **A** and **B**. Equal concentrations (45–90 μg) of WCE from each sample were subjected to immunoblotting with specific antibodies, as indicated. β-Actin served as a loading control. GIST, gastrointestinal stromal tumor; WCE, whole cell extract.

The effect of pharmacological inhibition of KIT/PDGFRA and Wee1 on cell cycle dynamics in GIST cells was measured with a BrdU assay. GIST-T1+Cas9 and GIST-T1+D842V KIT^KO^ cells treated with vehicle, MK-1775, avapritinib, or the combination were analyzed by flow cytometry after BrdU incorporation and subsequent antibody binding in combination with direct 7-AAD staining (Figure 5, A and B). MK-1775 treatment induced G_2_ phase cell cycle arrest in GIST-T1+Cas9 cells, whereas cells treated with avapritinib exhibited increased G0/G1-phase arrest compared with control cells. GIST-T1+Cas9 cells treated with the combination exhibited increased subG1 population, indicating increased apoptosis compared with either monotherapy treatment group (Figure 5A, top). Conversely, MK-1775 induced G0/G1-phase arrest in GIST-T1+D842V KIT^KO^ cells, whereas avapritinib induced G_2_ arrest compared with control cells. Combination treatment significantly increased the subG1 population compared with either monotherapy group (Figure 5A, bottom). The subG1 population was 2-fold higher in combination-treated GIST-T1+D842V KIT^KO^ cells compared with GIST-T1+Cas9 cells. To interrogate the mechanism of action of these inhibitors, we performed immunoblotting on GIST cell lines treated with MK-1775, avapritinib, or the combination (Figure 5C). After avapritinib treatment, inhibition of KIT and PDGFRA was observed in GIST-T1+Cas9 and GIST-T1+D842V KIT^KO^, respectively. Wee1 typically inhibits cell division cycle protein 2 (CDC2; also known as cyclin dependent kinase 1 [CDK1]) activity by phosphorylating it on 2 different sites, Tyr15 and Thr14, thereby decreasing its kinase activity and preventing entry into mitosis. Treatment with MK-1775 led to inhibition of Tyr15 on CDC2 (Figure 5C). Interestingly, both GIST-T1+Cas9 and GIST-T1+D842V KIT^KO^ cells treated with MK-1775 alone or in combination with avapritinib demonstrated increased γ-H2AX and cleaved-PARP, suggesting increased DNA double-strand breaks and apoptosis. We hypothesized that this increased DNA damage may be a result of loss of cell cycle checkpoints and decreased time for DNA repair mechanisms, ultimately causing increased cell death. Interestingly, the KIT-independent cell line, GIST-T1+D842V KIT^KO^, has significantly more cyclin D1 than the KIT-dependent line, GIST-T1+Cas9, in accordance with a recent report (37), providing a potential explanation for the differential effects of MK-1775 and avapritinib in these 2 cell lines.

### Combination treatment reduced tumor growth and improved survival in vivo.

**Figure 6:**
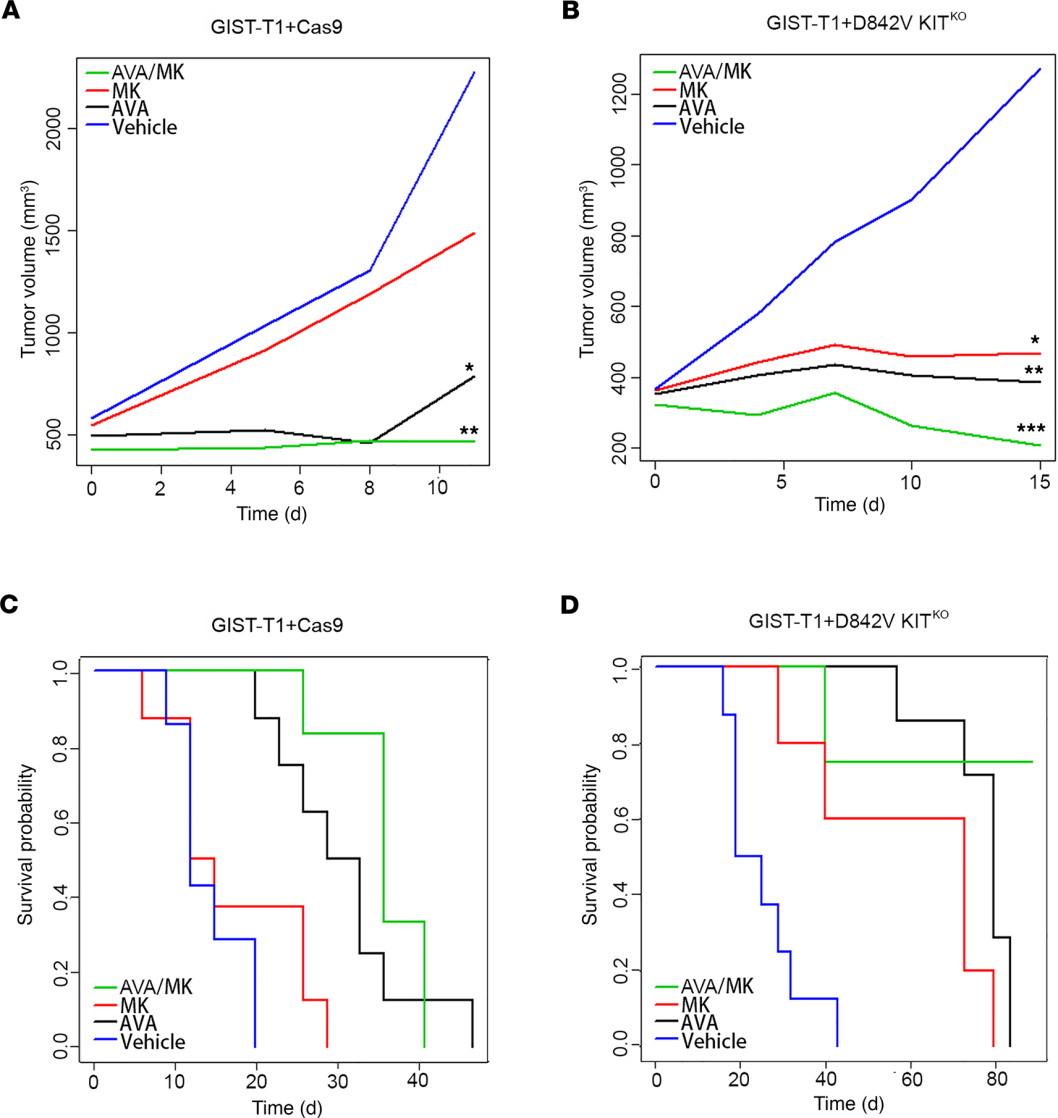
The combination of MK-1775 and avapritinib significantly inhibits GIST growth in vivo and improves disease-specific survival. (**A**) Statistically significant decreases in the rate of GIST-T1+Cas9 xenograft tumor growth were observed due to treatment with avapritinib (*P = 0.05, black) and avapritinib+MK-1775 combination (**P = 0.002, green) compared with vehicle group (blue) on day 11. (**B**) Statistically significant decreases in the rate of GIST-T1+D842V KIT^KO^ xenograft tumor growth were observed due to treatment with avapritinib (**P = 0.002) and MK-1775 (*P = 0.02) and avapritinib+MK-1775 (***P ≤ 0.0002) compared with vehicle group on day 15. Smoothed tumor growth curves (tumor volume vs. time) were computed for each treatment using the lowess smoother in the R statistical language. (**C**) Kaplan-Meier estimate of the probability of disease-specific survival of GIST-T1+Cas9 xenografts. Statistically significant differences (even after adjusting for multiple testing) in disease-specific survival were observed between the following comparisons: vehicle vs. avapritinib (*P* < 0.0001); vehicle vs. avapritinib/MK-1775 (*P* < 0.0001); and MK-1775 vs. avapritinib/MK-1775 (*P* < 0.0001). (**D**) Kaplan-Meier estimate of the probability of disease-specific survival of GIST-T1+D842V KIT^KO^ xenografts. Statistically significant differences (even after adjusting for multiple testing) in disease-specific survival were observed between the following comparisons: vehicle vs. MK-1775 (*P* = 0.01); vehicle vs. avapritinib (*P* < 0.0001); vehicle vs. avapritinib/MK-1775 (*P* < 0.0001); MK-1775 vs. avapritinib/MK-1775 (*P* = 0.01); and avapritinib vs. avapritinib/MK-1775 (*P* = 0.02). The overall test is also significant (*P* < 0.0001). GIST, gastrointestinal stromal tumor.

On the basis of these strong in vitro data, we hypothesized that there would be benefit in simultaneously inhibiting KIT/PDGFRA and Wee1, leading to loss of cell cycle checkpoint arrest, increased DNA damage, and ultimately increased cell death. To test this hypothesis, we performed a GIST xenograft study using the GIST-T1+Cas9 and GIST-T1+D842V KIT^KO^ cell lines. Xenografts were established subcutaneously in a total of 32 C.B17 SCID mice per cell line and randomized into 4 treatment arms: arm 1, vehicle; arm 2, MK-1775; arm 3, avapritinib; and arm 4, avapritinib/MK-1775 combination. GIST-T1+Cas9 xenografts showed disease stabilization in both the avapritinib monotherapy (*P* = 0.05) and avapritinib/MK-1775 combination (*P* = 0.002) arms compared with all other groups (Figure 6A). Significant disease stabilization was observed in GIST-T1+D842V KIT^KO^ xenografts in both avapritinib (*P* = 0.002) and MK-1775 (*P* = 0.02) monotherapy arms (Figure 6B). Combination-treated GIST-T1+D842V KIT^KO^ tumors showed disease stabilization and tumor regression (*P* ≤ 0.0002) on day 15 (Figure 6B). Importantly, GIST-T1+Cas9 tumor response led to significant improvement in disease-specific survival in the avapritinib/MK-1775 combination-treated group (*P* ≤ 0.0001) compared with vehicle group (Figure 6C). Kaplan-Meier curves for disease-specific survival of GIST-T1+D842V KIT^KO^ tumors demonstrated that avapritinib/MK-1775 combination-treated mice survived significantly longer than all other mice, including avapritinib alone (Figure 6D). Impressively, at the end of the study (89 days), 75% of the combination-treated mice were still alive, 1 without a measurable tumor, whereas no other vehicle and monotherapy-treated mice were alive. After treatment discontinuation, we observed regrowth of these tumors after approximately 4 weeks in all but one mouse, whose tumor never regrew.

## Discussion

Historically, treatment for advanced GIST involved the sequential application of IM, sunitinib, and regorafenib, regardless of genotype. This approach provided initial benefit to particular molecular subsets of GISTs (e.g., *KIT* mutants) and little to no benefit to others (e.g., *PDGFRA* D842V mutants). An increased understanding of GIST biology has revealed clear heterogeneity among the molecular subtypes and a corresponding need for novel therapeutics to target subtype-specific GISTs. Recently, this has been borne out with the success of avapritinib in the treatment of *PDGFRA* D842V mutant GISTs, prompting FDA approval of avapritinib as frontline therapy for this subtype in the unresectable or metastatic setting (12). Although the application of inhibitors targeting the primary mutant isoforms of KIT and PDGFRA has revolutionized the treatment of GIST, acquired resistance remains a serious clinical challenge. Addressing this challenge may require the identification and targeting of additional protein kinases within cancer-promoting cell signaling pathways that are active within GIST subtypes.

In this study, we utilized a chemical proteomics approach, a SILAC-based MIB-MS platform, to profile the kinome of human gastric GIST specimens along with normal gastric tissue. This platform provided a quantitative assessment of kinase abundance for nearly 60% of the human kinome. The kinomes of GIST primary tumors exhibit both a higher level of quantifiable kinases and a distinct profile compared with normal gastric tissues. This was not surprising because GISTs are generally characterized by gain-of-function mutations that activate multiple signaling pathways. Kinome profiling also revealed differences between RTK mutant (*KIT/PDGFRA)* GISTs and SDH-d GISTs that lack these mutations. This was expected given the distinct biology of KIT/PDGFRA-driven tumors and SDH-d GISTs (38, 39). Surprisingly, we also found that *PDGFRA* mutant GISTs expressed a distinct kinome pattern compared with *KIT* mutant tumors. Interestingly, these differences are partly due to elevated immune cell-associated kinases, including HCK, LCK, BTK, CSF1R, and MERTK. Two recent reports (27, 40) have used RNA-Seq to obtain immune profiles in GISTs. Vitiello et al. (27) profiled 75 GISTs (*n* = 37 *KIT* mutant and 24 *PDGFRA* mutant) and observed a notable increase in immune cells present in the *PDGFRA* cohort. Although Pantaleo et al. (40) did not report genotype specific differences in immune infiltrates in their cohort, their sample size was substantially smaller (*n* = 21 *KIT* mutant and 10 *PDGFRA* mutant), and some of these cases had IM treatment or were classified as unknown treatment status, which could potentially influence the number and activity of immune infiltrates.

Our GIST kinome profiling identified several well-studied and established kinases, such as KIT, PRKCQ (29), and FGFR1 (30), as significantly expressed kinases in all GISTs compared with normal tissue. In addition, our profiling identified other potential targets. We selected Wee1, gatekeeper of the G_2_/M cell cycle checkpoint, to evaluate because it was highly abundant in tumors compared with normal tissue and a largely understudied kinase in GISTs. Wee1 has been reported to be highly expressed in numerous malignancies including breast, hepatocellular, lung, melanoma, and others (33). To assess the role of Wee1, we utilized the Wee1 inhibitor MK-1775 (adavosertib, AZD1775), which has been evaluated in numerous preclinical and clinical trials as single agent or in combination, often with DNA damaging agents (41–43). Notably, recent reports have highlighted synergistic potential for MK-1775 in combination with other kinase inhibitors, including TAK228 (34) and alisertib (35). Our loss-of-function studies targeting Wee1 in an isogenic pair of cell lines driven by KIT (GIST-T1+Cas9) or PDGFRA (GIST-T1+D842V KIT^KO^) revealed an essential role for Wee1 in GIST cell proliferation, suggesting Wee1 as a plausible drug target in GISTs. We demonstrated enhanced drug combination effects between avapritinib and MK-1775 in both KIT and PDGFRA-driven cell lines using two-dimensional and 3D in vitro viability studies. Whereas additive effects of the combination were observed in GIST-T1+Cas9 cells, strong synergy was observed in GIST-T1+D842V KIT^KO^ cells treated with the combination. BrdU assays indicated differences in the effects of both MK-1775 and avapritinib on cell cycle between the 2 cell lines, and enhanced apoptosis in the PDGFRA-driven cell line compared with its KIT-driven counterpart. We believe that these differences are due in part to differential expression of cyclin D1, a regulator of the G1/S cell cycle checkpoint, which was recently identified as an oncogenic mediator in KIT-independent GISTs (38). Increased expression of γH2AX suggests that increased DNA damage, most likely due to loss of cell cycle checkpoint, is responsible for enhanced cell death in combination-treated cells.

The results of these in vitro studies provided justification for investigating such an approach in vivo to determine whether this combination would improve efficacy of avapritinib and/or increase time to resistance in GIST xenografts. Similar to the in vitro studies, the avapritinib+MK-1775 combination was significantly better at repressing tumor growth compared with both single agents in both xenograft models; however, tumor regression was observed only in the GIST-T1+D842V KIT^KO^ line. Interestingly, MK-1775 alone had a considerable effect on tumor volume compared with vehicle in only the PDGFRA-driven xenografts, indicating inherent cell cycle differences in KIT-driven versus PDGFRA-driven GISTs. These differences were most noticeable when examining disease-specific survival. Impressively, at the end of the study (89 days), in the combination treated arm, 75% of the mice were alive, whereas no other mice, including the avapritinib monotherapy group, survived. Together, these xenograft studies provide strong evidence to support future clinical studies evaluating the use of avapritinib in combination with Wee1 inhibitors in patients with *PDGFRA* mutant GISTs and IM-refractory *KIT* mutant GISTs.

During the preparation of this manuscript, Liu et al. (44) published a report examining Wee1 in GISTs. They reported elevated expression of Wee1 in GISTs compared with normal gastric tissues and an antiproliferative effect of Wee1 knockdown and MK-1775 treatment with DNA damage induction and increased apoptosis. These findings concur with our findings. However, their studies involved *KIT* mutant GISTs only. Our work indicates that in addition to *KIT* mutant GIST, Wee1 may be a more promising target in *PDGFRA* mutant GISTs. We also hypothesized that Wee1 could be a target in SDH-d GISTs based on our kinome profiling data and its independence of KIT. Furthermore, we expanded our analysis to include not only *PDGFRA* mutant GIST cell lines but also in vivo studies of MK-1775, whereas Liu et al. limited studies to in vitro evaluations of MK-1775 in *KIT* mutant GISTs. Therefore, our work underscores and expands the evidence for Wee1 serving an important role in GIST biology and provides a strong rationale for the therapeutic targeting of Wee1 in all subtypes of GIST.

## Methods

*Kinome profiling experimental design, data analysis, and statistical rationale*. For proteomic measurement of kinase abundance in tissues, we used MIB-MS profiling and quantitated kinase levels using a combination of LFQ and s-SILAC (22, 23). Briefly, an equal amount of s-SILAC reference (5 mg) was spiked into each primary tissue sample (5 mg); kinases were purified from tissues using MIB-resins, eluted, and digested; and peptides were analyzed by LC-MS/MS as previously described (19). To identify differences in kinase abundance among GIST tissues, we performed MIB-MS analysis on GIST (*n* = 33; *KIT* mutant, *n* = 15; *PDGFRA* mutant, *n* = 10; SDH-d, *n* = 8) and normal gastric tissues (*n* = 9). Measurement of MIB-enriched kinase abundance in tissues was performed by LFQ and s-SILAC quantitation using MaxQuant software version 1.6.1.0.

### Data analysis of MIB-MS

MaxQuant-normalized LFQ values or SILAC ratios (H/L) were filtered for human protein kinases in Excel and then imported into Perseus software (1.6.2.3) for quantitation.

#### LFQ data processing.

Kinase LFQ values were filtered in the following manner: kinases identified by site only were removed, and reverse or potential contaminants were removed and then filtered for kinases identified by >1 unique peptide. Kinase LFQ intensity values were then log2-transformed, technical replicates were averaged, and rows were filtered for minimum valid kinases measured (*n* ≥ 70% of runs). No imputation of missing values was performed. Filtered LFQ data were annotated and subjected to a Student’s *t* test comparing GIST tissue subtypes using Perseus software. Parameters for the Student’s *t* test were as follows: S0 = 0.1, side both using Permutation-based FDR <0.05.

#### s-SILAC data processing.

Kinase s-SILAC ratios were transformed 1/(x) to generate light/heavy ratios and log2-transformed; technical replicates were averaged; and rows were filtered for minimum valid kinases measured (*n* ≥ 70% of runs). No imputation of missing values was performed. Filtered normalized s-SILAC ratios were annotated and subjected to a Student’s *t* test comparing GIST tissue subtypes using Perseus software. Parameters for the Student’s *t* test were as follows: S0 = 0.1, side both using Permutation-based FDR <0.05. Volcano plots depicting differences in kinase abundance were generated using RStudio software. For PCA analysis of kinase log2 LFQ values, rows were filtered for kinases measured in 100% of MIB-MS runs, and PCA (PC1 vs. PC2, PC2 vs. PC3, and PC1 vs. PC3) was performed to visualize kinome profiles among tissue samples. For hierarchical clustering (Euclidean) of kinase levels among tissue samples, MIB-enriched kinase log2 LFQ intensities were *z* score–normalized in Perseus, followed by row filtering for minimum valid kinases measured (*n* ≥ 70% of runs). Scatter plots or bar graphs were used to compare LFQ versus s-SILAC measurements of differentially expressed kinases among tumor and normal tissues. Plots comparing differences in kinase log2 LFQ values or kinase log2 s-SILAC ratios were determined by Student’s *t* test. Scatter plots depicting differences in kinase abundance were generated using RStudio software and bar graphs generated in Excel or Prism.

#### Nano–LC-MS/MS.

Proteolytic peptides were resuspended in 0.1% formic acid and separated with a Thermo Scientific RSLC Ultimate 3000 on a Thermo Scientific Easy-Spray C18 PepMap 75 μm × 50 cm C-18 2 μm column. For MIB runs, a 240-minute gradient of 4%–25% acetonitrile with 0.1% formic acid was used. For total proteome runs, a 305-minute gradient of 2%–20% (180 minutes), 20%–28% (45 minutes), and 28%–48% (20 minutes) acetonitrile with 0.1% formic acid was used. Both gradients were run at 300 nL/minute at 50^o^C. Eluted peptides were analyzed by Thermo Scientific Q Exactive or Q Exactive plus MS utilizing a top 15 methodology, in which the 15 most intense peptide precursor ions were subjected to fragmentation. The AGC for MS1 was set to 3 × 10^6^ with a max injection time of 120 minutes; the AGC for MS2 ions was set to 1 × 10^5^ with a max injection time of 150 minutes; and the dynamic exclusion was set to 90 seconds.

#### Proteomics data processing.

Raw data analysis of LFQ or s-SILAC experiments was performed using MaxQuant software 1.6.1.0 and searched using Andromeda 1.5.6.0 against the Swiss-Prot human protein database (downloaded on April 24, 2019; 20,402 entries). The search was set up for full tryptic peptides with a maximum of 2 missed cleavage sites. All settings were default and searched using acetylation of protein N-terminus and oxidized methionine as variable modifications. Carbamidomethylation of cysteine was set as fixed modification. The precursor mass tolerance threshold was set at 10 ppm and maximum fragment mass error was 0.02 Da. LFQ quantitation was performed using MaxQuant with the following parameters. LFQ minimum ratio count: 2, Fast LFQ: selected, LFQ minimum number of neighbors; 3, LFQ average number of neighbors: 6. SILAC quantification was performed using MaxQuant by choosing multiplicity as 2 in group-specific parameters and Arg10 and Lys8 as heavy labels.

Global parameters for protein quantitation were as follows: label minimum ratio count: 1, peptides used for quantitation: unique, only use modified proteins selected and with normalized average ratio estimation selected. Match between runs was employed for LFQ and s-SILAC quantitation and the significance threshold of the ion score was calculated based on a FDR of <1%.

#### MIBs preparation and chromatography.

Experiments using MIB/MS were performed as previously described (*25*). Briefly, cells or tumors were lysed and an equal amount of the s-SILAC reference (5 mg) lysate was added to nonlabeled (5 mg) lysate (cell or tumor tissue) and endogenous kinases isolated by flowing lysates over kinase inhibitor–conjugated Sepharose beads (purvalanol B, VI16832, PP58, and CTx-0294885 beads) in 10 mL gravity-flow columns. Eluted kinases were reduced by incubation with 5 mM DTT at 65°C for 25 minutes and alkylated with 20 mM iodoacetamide at room temperature for 30 minutes in the dark. Alkylation was quenched with DTT for 10 minutes, flowed by digested with sequencing-grade modified trypsin (Promega) overnight at 37°C. C-18 purified peptides were dried in a speed vacuum, and subsequent LC-/MS/MS analysis was performed.

#### Cell lines, compounds, and antibodies.

GIST-T1 tumor cell line possessing a heterozygous mutation in *KIT* exon 11 was provided by Takahiro Taguchi (Kochi University, Kochi, Japan) (45). GIST-T1+Cas9 and GIST-T1+D842V KIT^KO^ are sublines of GIST-T1. GIST-T1+Cas9 was generated transduction of Cas9 using the LentiV-Cas9-Puro vector system provided by Christopher Vakoc (Cold Spring Harbor Laboratory, Cold Spring, New York). GIST-T1+D842V KIT^KO^ subline was created by transducing cells with D842V mutant PDGFRA. Endogenous KIT expression was knocked out using CRISPR/Cas9. Knockout was verified at protein and DNA levels. All GIST-T1 cell lines were grown in Iscove’s Modified Dulbecco’s Media with 15% FBS and were routinely monitored by Sanger sequencing to confirm *KIT*/*PDGFRA* mutation status and cell identity. The GIST882 tumor cell line possessing a homozygous mutation in *KIT* exon 13, provided by Jonathan A. Fletcher (Dana Farber Cancer Institute, Boston, Massachusetts, USA), was grown in RPMI with 15% FBS. Cell lines were regularly tested for mycoplasma contamination by PCR and MycoAlert Mycoplasma Detection Kit (Lonza). Avapritinib and MK-1775 were obtained from Selleckchem. For in vitro experiments, avapritinib and MK-1775 were dissolved in DMSO. For in vivo experiments, avapritinib and MK-1775 were dissolved in 2% DMSO plus 40% PEG400 plus 2% Tween80 plus ddH_2_O. The following antibodies were purchased from Cell Signaling Technology: PDGFRA (3174), phospho-PDGFRA (3170S), c-KIT (3392S), phospho-c-KIT (3391S), phospho-cdc-2 (9111), Cdc-2 (9116S), PARP (9542), Cleaved-PARP (5625S), and Cyclin D1 (2978T). Wee1 antibody (ab111820) was purchased from Abcam. β-Actin (A5441) and γ-H2AX (05-636) antibodies were purchased from MilliporeSigma.

#### siRNA transfection.

The custom siRNA library was synthesized with 4 independent siRNAs pooled per target (siGenome SMARTpool, Dharmacon). Transfection conditions were determined for GIST-T1+Cas9 and GIST-T1+D842V KIT^KO^ cells using siRNA SMARTpools against KIT, PDGFRA, and GL-2 (Dharmacon) controls to achieve Z′ factor of 0.5 or greater. Reverse transfection mixtures were assembled in 96-well plates with final siRNA concentration of 50 nM. After 72 hours, plates were assayed for cell viability using the CellTiter Blue (CTB) Viability Assay (Promega) as previously described (46).

#### Cell proliferation/viability assay.

Tumor cells were plated in 96-well plates at optimal 0.6 × 10^4^ densities and incubated overnight. Wells were treated in sextuplicate with varying doses of MK-1775 and/or avapritinib. Cell proliferation and viability were measured at 72 hours after treatment using CTB Assay as described above. Assays were performed as 3 independent biological replicates, with a minimum of 3 technical replicates in each treatment arm. An increasing dose series was used for each drug to estimate LD50. A function of form *A* + (1 – *A*) × exp(–*B* × dose) or *A* + (1 – *A*)/[1 + (dose/*B*)*^p^*] was fit to data by least squares, where *A* denotes the survival fraction of cells at extremely high doses (in both formulae); *B* denotes kill rate (in the first formula) or the dose level giving survival fraction halfway between level *A* and 1 (in the second formula); and *p* denotes the power of (dose/*B*) that determines the steepness of the sigmoidal curve at its inflection point. These functions were used to interpolate surviving fractions between those in dose series and set to one-half to estimate corresponding LD50s (LD50-1 and LD50-2). Increasing series of combination doses in the same ratio were used as their LD50s to estimate LD50 of that combination (dose 1 and dose 2), using an interpolating function. If the CI = dose 1/LD50-1 + dose 2/LD50-2 < 1, then the point (dose 1, dose 2) may be synergistic; otherwise, it was considered either additive or antagonistic. If not considered an additive or antagonistic, a bootstrap resampling method was used to test the null hypothesis of no synergism (36).

#### Spheroid drug sensitivity.

Spheroids were formed in 96-Well U-Bottom Clear Cell Repellent Surface Microplates (Greiner Bio-One). GIST-T1+Cas9, GIST-T1+D842V KIT^KO^, and GIST882 cells were suspended in complete media (4500 cells/well) for 24 hours for spheroid formation. Spheroids were treated with appropriate drug(s) and were imaged (original magnification, ×4) by EVOS FL Digital Inverted Microscope after 120 hours of treatment. Spheroid surface area and viability were measured and statistical analyses were conducted as described previously (46). Three independent biological replicate experiments were performed with minimum of 3 technical replicates in each treatment arm.

#### BrdU incorporation assay.

The DNA synthesis proliferation rate was measured using BrdU Flow Kit (BD Biosciences) according to manufacturer’s protocol. Treated GIST-T1+Cas9 and GIST-T1-D842V+KIT^KO^ cells were labeled with BrdU for 3.5 hours. Anti-FITC-BrdU antibody was used in GIST-T1+Cas9 cells and anti-APC-BrdU was used in GIST-T1-D842V+KIT^KO^ cells. Total DNA was stained with 7-amino-actinomycin D (7-AAD). Double-labeled samples were analyzed using 2-color flow cytometric analysis conducted on LSR ll BD Flow Cytometer. Data were analyzed and displayed using FlowJo software.

#### Preparation of whole cell extract from cells and immunoblot assays.

The whole cell extracts were prepared and evaluated by immunoblot assay as previously described (47).

#### GIST xenografts and drug administration.

GIST-T1+Cas9 and GIST-T1+D842V KIT^KO^ cells were washed and resuspended in PBS at a density of 1 × 10^6^ cells/100 μL. Cells in PBS (100 μL) were mixed thoroughly with Matrigel Matrix (100 μL; BD Biosciences) and suspension was injected subcutaneously into the right flanks of 8- to 9-week-old (female or male) SCID mice (CB.17/SCID, Taconic Biosciences). A total of 65 mice were used in this study, with all treatment groups having 7 or more mice. Tumor volume was calculated as previously described (46). When tumors reached approximately 300 mm^3^, mice were randomized into 4 treatment arms: arm 1, vehicle; arm 2, MK-1775 at 60 mg/kg, twice per day (oral); arm 3, avapritinib 10 mg/kg, once per day (oral); and arm 4, combination of MK-1775 and avapritinib at monotherapy doses. Treatment was continued until tumors exceeded 10% of their body weight or animals demonstrated distress or weight loss greater than 10%.

#### Tumor growth modeling.

Tumor volume was measured for every mouse in all treatment arms (vehicle, MK-1775, avapritinib, and combination) at a total of 15 distinct time points in GIST-T1+Cas9 xenografts, from baseline (day 0) until study conclusion (47 days) and 25 distinct time points in GIST-T1+D842V KIT^KO^ xenografts, from baseline (day 0) until study conclusion (day 89). A longitudinal model based on generalized estimating equations approach (Gaussian model with identity link and autoregressive correlation structure) was used to model treatment effect and time on (the logarithm of) tumor volume. A linear time effect was included in the model for logarithm of tumor volume and interacted with treatment. Disease-specific survival and tumor volume were compared between treatment groups using log rank and Mann-Whitney *U* tests, respectively. All tests were 2-sided and used a type I error of 5%. The package geepack and survival in R statistical language and environment was used in these computations.

#### Data availability.

All MS proteomics data have been deposited to the ProteomeXchange Consortium via PRIDE partner repository with the data set identifier PXD020720.

#### Statistics.

Statistical analyses were performed using Graph Pad Prism 5.0. Data are shown as mean ± SD or SEM. Data were reported as biological replicates, with technical replicates indicated in the figure legends. One-way ANOVA was performed in spheroid assay. Student *t* tests (unpaired 2-tailed) were performed in BrdU assay. Regression analysis (R^2^) among MIB-MS quantitative method was performed in Perseus software. Smoothed tumor growth curves (tumor volume vs. time) were computed for each treatment using the lowess smoother in the R statistical language. Kaplan-Meier was used to estimate disease-specific survival of mice. A *P* value of less than 0.05 was considered significant.

#### Study approval.

All studies involving animals were reviewed and approved by the Fox Chase Cancer Center Institutional Animal Care and Use Committee. Patient sample collection and analysis were conducted following a protocol approved by an institutional review board at Fox Chase Cancer Center. Written informed consent was obtained from patients for use of samples.

## Author contributions

SY, LR, JSD, and MVM conceptualized the research, designed the experiments, and wrote the manuscript. SY, DS, MK, JD, MGB, and MBE performed experiments and analyzed data. KJL, KD, YZ, and SL performed statistical analyses. LK and MCH provided *KIT* mutant and PDGFRA mutant GIST cell lines. RDM provided GIST samples.

## Supplementary Material

Supplemental data

Supplemental Data Set 1

## Figures and Tables

**Table 1:**
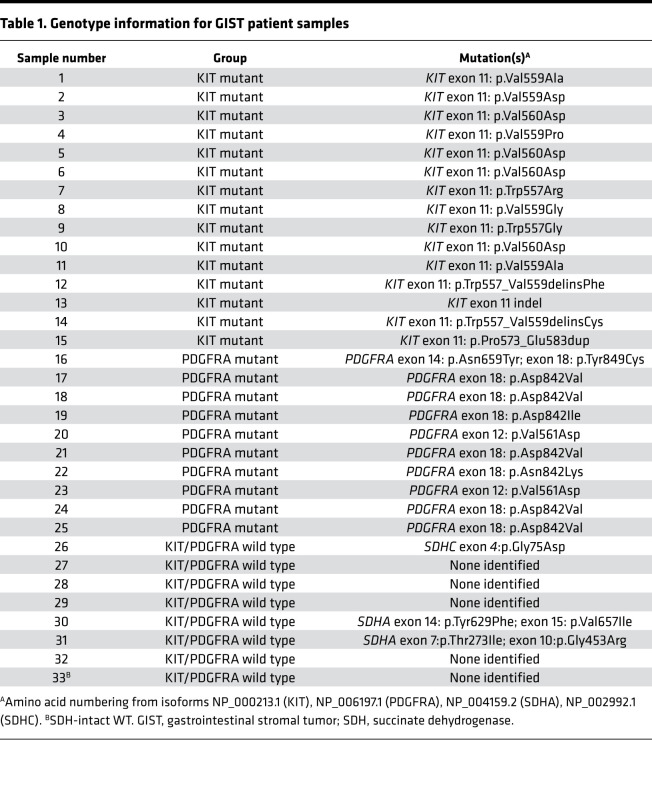
Genotype information for GIST patient samples

## Version 1 12/15/2020

In-Press Preview

## Version 2 01/25/2021

Electronic publication
