# Supplementary material for: Identification of Wee1 as a target in combination with avapritinib for gastrointestinal stromal tumor treatment
Source: JCI Insight. 2021 Jan 25;6(2):e143474. doi: 10.1172/jci.insight.143474 (PMC7934848; doi:10.1172/jci.insight.143474)
Supplement: Supplemental data [file jciinsight-6-143474-s164.pdf]

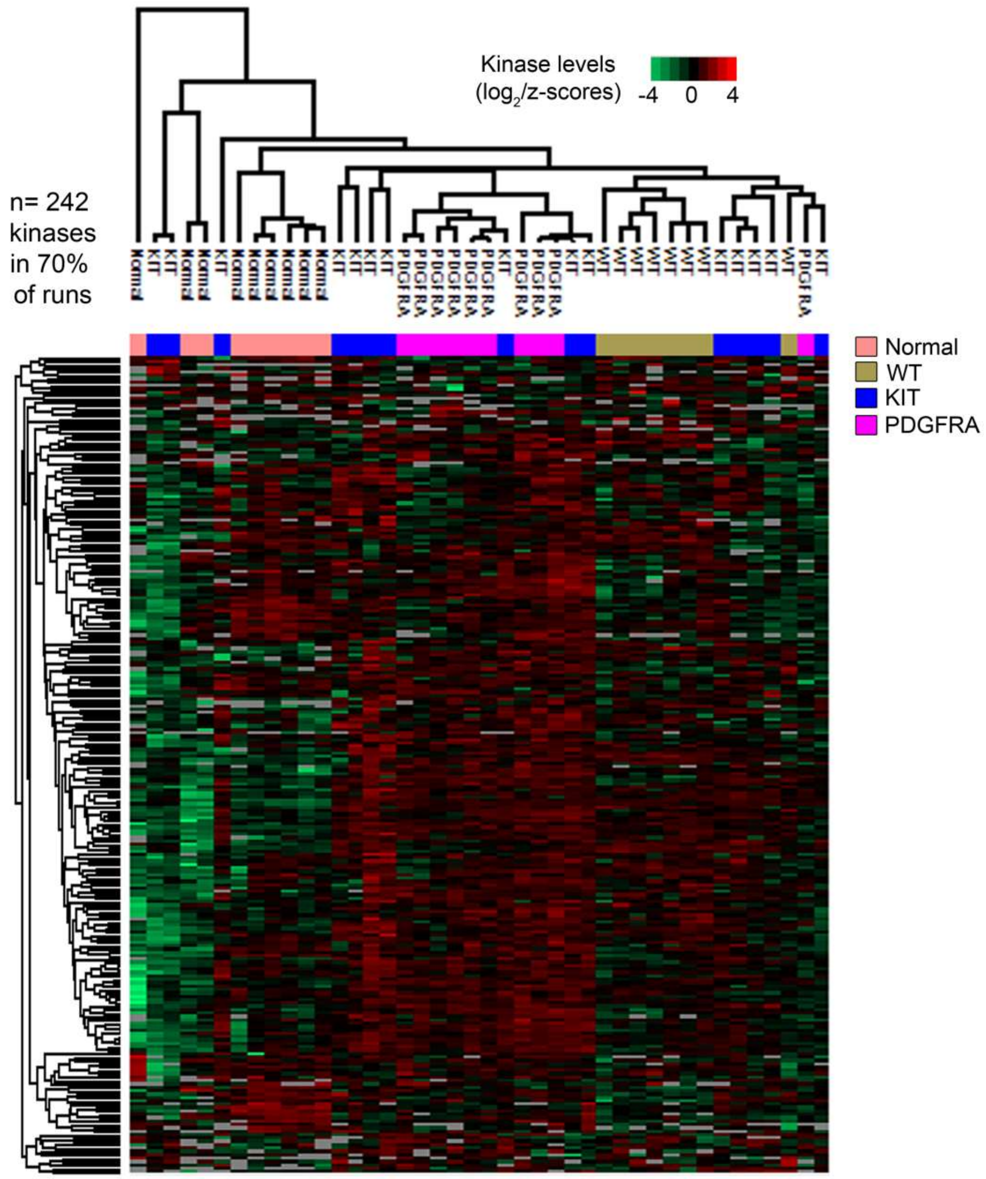

Supplemental Figure 1. Hierarchical clustering of kinase log2 LFQ z-scores of WT, KIT-mutant, PDGFRA-mutant tumors and normal gastric tissues, as determined by MIB-MS.

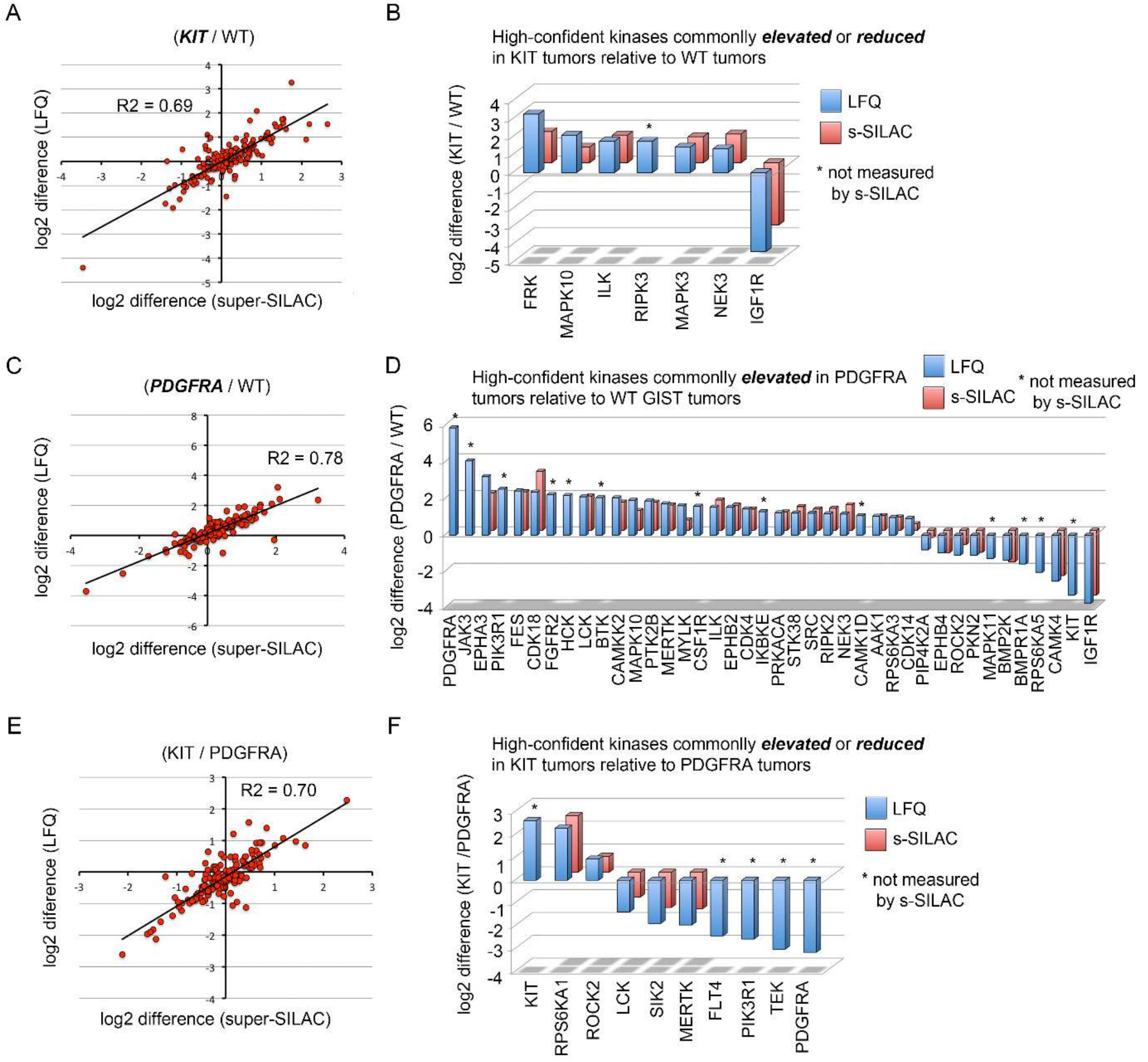

Supplemental Figure 2. Defining kinome signatures of GIST tumor subtypes using MIB-MS profiling (**A**, **C** and **E**) Scatterplot depicts the overlap in kinases elevated or reduced determined by LFQ or s-SILAC comparing (A) *KIT*-mutant vs WT, (C) *PDGFRA*-mutant vs WT and (E) *KIT*-mutant vs. *PDGFRA*-mutant. Regression analysis (R^2) among quantitative methods was performed in Perseus Software. Differential expressed kinases commonly identified by LFQ and s-SILAC quantitation (FDR <0.05) are labeled. (**B**, **D** and **F**) Bar graph depicts high-confident kinases log2 LFQ values overexpressed in (B) *KIT*-mutant vs. WT, (D) *PDGFRA*-mutant vs. WT or (F) *KIT*-mutant vs *PDGFRA*-mutant GIST primary tumors determined by LFQ and/or s-SILAC quantitation (FDR <0.05).

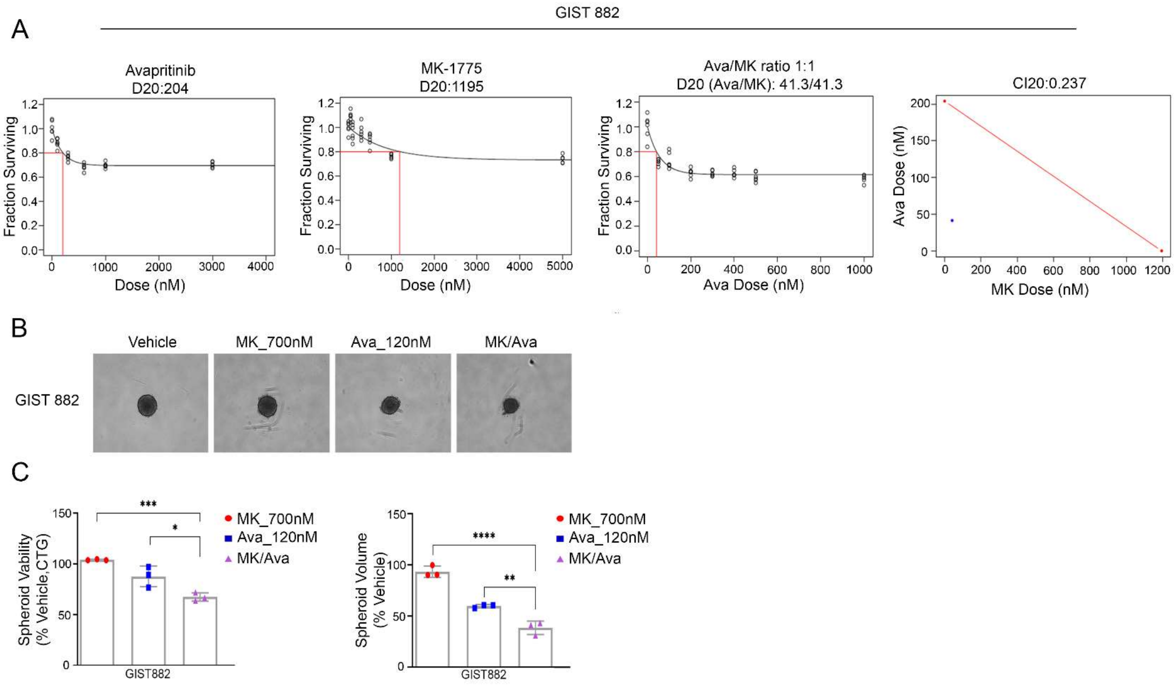

Supplemental Figure 3. MK-1775 and avapritinib have enhanced combination on in vitro GIST882 cell growth.

(A) Panels 1 & 2, Dose response curves for single agents (avapritinib, MK-1775) in GIST882 cell line. Red box indicates estimation of LD20 concentration for each single drug. **Panel 3**, Dose response curve representing increasing series of combinations in GIST882 cell line. Red box indicates estimation of LD20 concentration for combination of drugs. **Panel 4,** Single point (blue) on isobole curve for 20% kill. Red line indicates 50% isobole for strictly additive effect. CI_{LD20} in GIST882 is 0.0237 and is found within the synergistic triangle (region below the red line). **(B)** Representative images of GIST882 spheroids after 120-hour treatment at indicated concentrations. **(C, left panel)** Bars represent average viability + SEM after 120-hour treatment at indicated drug concentrations for GIST882 spheroids as a percentage of vehicle-treated spheroids**. (C, right panel)** Bars represent the average spheroid volume + SEM of GIST882 spheroids as a percentage of vehicle-treated spheroids. All spheroid data were analyzed using GraphPad Prism, with comparisons of treatment groups performed in one-way ANOVA and post hoc comparisons made using Bonferroni multiple comparisons method * p = 0.0249, ** p =0.0042, *** p = 0.0006, **** p =< 0.0001.
